# Comparison of five glomerular filtration rate estimating equations as predictors of acute kidney injury after cardiovascular surgery

**DOI:** 10.1038/s41598-019-47559-w

**Published:** 2019-07-30

**Authors:** Jun-Young Jo, Seung Ah Ryu, Jong-Il Kim, Eun-Ho Lee, In-Cheol Choi

**Affiliations:** 1 Department of Anaesthesiology and Pain Medicine, Laboratory for Perioperative Outcomes Analysis and Research, Asan Medical Centre, University of Ulsan College of Medicine, Seoul, Korea; 2 Department of Anaesthesiology and Pain Medicine, Seoul Medical Centre, Seoul, Korea

**Keywords:** Outcomes research, Acute kidney injury

## Abstract

We aimed to compare the ability of preoperative estimated glomerular filtration rate (eGFR), calculated using five different equations, to predict adverse renal outcomes after cardiovascular surgery. Cohorts of 4,125 adult patients undergoing elective cardiovascular surgery were evaluated. Preoperative eGFR was calculated using the Cockcroft-Gault, Modification of Diet in Renal Disease (MDRD) II, re-expressed MDRD II, Chronic Kidney Disease Epidemiology Collaboration, and Mayo quadratic (Mayo) equations. The primary outcome was postoperative acute kidney injury (AKI), defined by Kidney Disease: Improving Global Outcomes Definition and Staging criteria based on changes in serum creatinine concentrations within 7 days. The MDRD II and Cockcroft-Gault equations yielded the highest (88.1 ± 26.7 ml/min/1.73 m^2^) and lowest (79.6 ± 25.5 ml/min/1.73 m^2^) mean eGFR values, respectively. Multivariable analysis showed that a preoperative decrease in renal function according to all five equations was independently associated with an increased risk of postoperative AKI. The area under the receiver operating characteristics curve for predicting postoperative AKI was highest for the Mayo equation (0.713). Net improvements in reclassification and integrated discrimination were higher for the Mayo equation than for the other equations. The Mayo equation was the most accurate in predicting postoperative AKI in patients undergoing cardiovascular surgery.

## Introduction

Preoperative renal dysfunction is prevalent in patients undergoing cardiovascular surgery, and is an independent risk factor for morbidity and mortality after surgery^1–3^. However, despite its clinical importance, preoperative evaluation of renal function is often underused resources for clinicians.

The level of glomerular filtration rate (GFR), which is generally accepted as the best overall indicator of renal function, is vital to the detection of renal dysfunction, understanding its severity, and making decisions about diagnosis, prognosis, and treatment. However, because of the impracticality of direct measurement of GFR in daily clinical practice, it has been recommended to use estimated GFR (eGFR) calculated from several equations incorporating variables such as serum creatinine (sCr), age, weight, and sex^4^. Thus, early detection and evaluation of patients with pre-existing renal dysfunction using eGFR may allow appropriate modification of perioperative care and ultimately improve postoperative outcomes. To date, several equations were developed to calculate eGFR. The commonly used equations to calculate eGFR include the Cockcroft-Gault (CG) and Modification of Diet in Renal Disease (MDRD) equation^5,6^. Other equations used to calculate eGFR include the re-expressed MDRD II equation, developed for use with standardized sCr assays^7^; the Mayo Quadratic (Mayo) equation, developed to better estimate GFR in healthy patients with preserved renal function^8^; and the recently developed Chronic Kidney Disease Epidemiology Collaboration (CKD-EPI) equation^9^. Of these five equations, CKD-EPI equation is known to have the highest accuracy in estimating GFR and is currently recommended for use to estimate GFR by the 2012 Kidney Disease: Improving Global Outcomes (KDIGO) clinical practice guidelines^10^. However, in addition to accurately estimating GFR, the prognostic ability of these equations may also be clinically important. Indeed, the prognostic values of these equations have been compared in various medical settings, including in patients with heart failure and myocardial infarction^11–16^. To our knowledge, however, no study has compared the ability of these equations to predict outcomes after cardiovascular surgery.

The aim of this study therefore was to evaluate the agreement between eGFRs calculated using five different equations based on a single preoperative sCr value, as well as to confirm which equation can best predict postoperative renal dysfunction in patients undergoing cardiovascular surgery.

## Results

**Figure 1:**
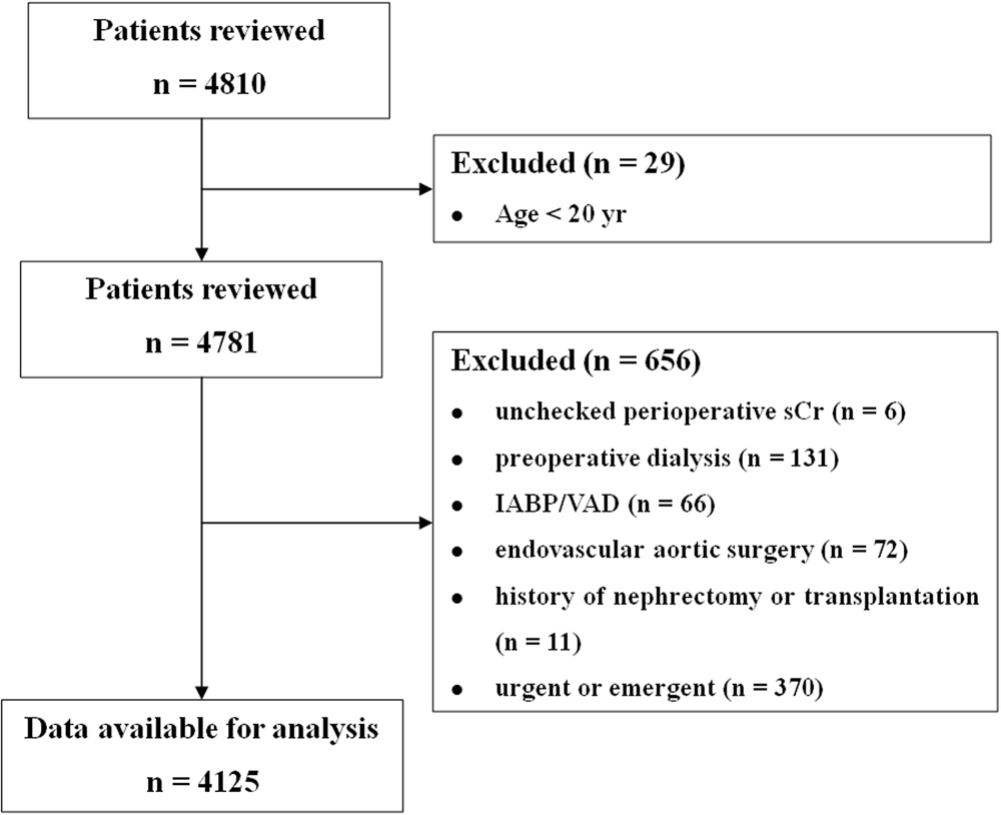
Study inclusion/exclusion flow diagram.

Of the 4,810 patients who underwent cardiovascular surgery within the study period, 685 were excluded, and 4,125 were included in this analysis (Fig. 1). Table 1 shows the baseline and perioperative characteristics of the study population. Their mean age was 60.6 ± 12.4 years; 60.2% were male; and their mean preoperative sCr level was 0.9 ± 0.5 mg/dl. Of these 4,125 patients, 1,256 (30.4%) developed acute kidney injury (AKI) after cardiovascular surgery, including 417 (10.1%) who developed KDIGO stage ≥2 and 219 (5.3%) who required renal replacement therapy.

**Table 1:** Baseline and perioperative characteristics of the patient population.

Variables	Missing	Total	AKI	No AKI	*P* value
N		4125	1256	2869	
Baseline characteristics					
Male gender (n, %)	0	2483 (60.2)	734 (58.4)	1749 (61.0)	
Age (yr)	0	60.6 ± 12.4	63.2 ± 11.7	59.4 ± 12.5	<0.001
<40 yr		272 (6.6)	51 (4.1)	221 (7.7)	
40–49 yr		446 (10.8)	106 (8.4)	340 (11.9)	
50–59 yr		1013 (24.6)	257 (20.5)	756 (26.4)	
60–69 yr		1289 (31.2)	399 (31.8)	890(31.0)	
≥70 yr		1105 (26.8)	443 (35.3)	662 (23.1)	
BMI (kg/m^2^)	1	24.0 ± 3.3	23.9 ± 3.5	24.0 ± 3.3	0.231
EuroSCORE (logistic)	0	3.6 [1.9–6.8]	4.5 [2.3–9.0]	3.1 [1.7–6.0]	<0.001
Haematocrit (%)	0	38.8 [35.2–41.9]	37.1 [33.3–40.4]	39.3 [36.0–42.4]	<0.001
Creatinine (mg/dl)	0	0.9 ± 0.5	1.0 ± 0.5	0.9 ± 0.5	<0.001
Bilirubin, total (mg/dl)	1	0.8 ± 0.5	0.9 ± 0.6	0.7 ± 0.4	<0.001
Albumin (g/dl)	1	3.8 ± 0.5	3.6 ± 0.5	3.8 ± 0.4	<0.001
Uric acid (mg/dl)	2	5.7 ± 1.8	6.0 ± 2.0	5.6 ± 1.7	<0.001
LVEF (%)	3	60 [54–64]	59 [52–64]	60 [55–65]	<0.001
Diabetes mellitus	0	998 (24.2)	356 (28.3)	642 (22.4)	<0.001
Hypertension	0	2058 (49.9)	713 (56.8)	1345 (46.9)	<0.001
Congestive heart failure	0	316 (7.7)	129 (10.3)	187 (6.5)	<0.001
Previous MI	0	253 (6.1)	69 (5.5)	184 (6.4)	0.288
Cerebrovascular disease	0	450 (10.9)	178 (14.2)	272 (9.5)	<0.001
PVD	0	475 (11.5)	190 (15.1)	285 (9.9)	<0.001
Liver disease	0	222 (5.4)	85 (6.8)	137 (4.8)	0.011
COPD	0	143 (3.5)	45 (3.6)	98 (3.4)	0.859
Dyslipidemia	0	2839 (68.8)	782 (62.3)	2057 (71.7)	<0.001
Smoker, current	0	795 (19.3)	241 (19.2)	554 (19.3)	0.961
ACEI or ARB	0	1931 (46.8)	653 (52.0)	1278 (44.5)	<0.001
β-blocker	0	1918 (46.5)	647 (51.5)	1271 (44.3)	<0.001
CCB	0	1979 (48.0)	655 (52.1)	1324 (46.1)	<0.001
Diuretics	0	1719 (41.7)	591 (47.1)	1128 (39.3)	<0.001
Insulin	0	417 (10.1)	175 (13.9)	242 (8.4)	<0.001
OHA	0	779 (18.9)	279 (22.2)	500 (17.4)	<0.001
Aspirin	0	1703 (41.3)	521 (41.5)	1182 (41.2)	0.893
Clopidogrel	0	968 (23.5)	271 (21.6)	697 (24.3)	0.064
Statins	0	2025 (49.1)	611 (48.6)	1414 (49.3)	0.731
Intraoperative data					
Type of surgery					
CABG	0	1228 (29.8)	319 (25.4)	909 (31.7)	
Valve	0	1856 (45.0)	550 (43.8)	1306 (45.5)	
Aorta	0	264 (6.4)	107 (8.5)	157 (5.5)	
Combined	0	777 (18.8)	280 (22.3)	497 (17.3)	
Off-pump surgery	0	1029 (24.9)	265 (21.1)	764 (26.6)	
Operation time (min)	0	306.6 ± 108.2	342.5 ± 134.8	290.8 ± 89.8	<0.001
CPB time (min)	0	110.7 ± 86.5	132.6 ± 99.1	101.1 ± 78.4	<0.001
Total crystalloid (L)	0	1.6 [1.2–2.2]	1.5 [1.1–2.1]	1.6 [1.2–2.2]	0.024
Total colloid (L)	0	0.5 [0.5–1.0]	0.7 [0.5–1.0]	0.5 [0.5–1.0]	<0.001

Data are expressed as number of patients (%), mean ± standard deviation, or median [first-third quartiles].

BMI, body mass index; EuroSCORE, European System for Cardiac Operative Risk Evaluation; LVEF, left ventricle ejection fraction; MI, myocardial infraction; PVD, peripheral vascular disease; COPD, chronic obstructive pulmonary disease; ACEI, angiotensin-converting enzyme inhibitor; ARB, angiotensin receptor blocker; CCB, calcium channel blocker; OHA, oral hypoglycemic agent; CABG, coronary artery bypass grafting; CPB, cardiopulmonary bypass.

The correlation and Bland-Altman analyses between 4 equations and the CKD-EPI equation as the reference are shown in Supplementary Fig. S1. The correlation analyses showed a significant and strong correlation between 4 equations and the CKD-EPI equation, especially at a low eGFR. Bland-Altman analyses also showed a relatively good agreement between the values of the 4 equations and the value of the CKD-EPI equation only when the eGFR was low. However, the higher the eGFR value, the larger the difference between the 4 equations and the CKD-EPI equation. Also, except for the Mayo equation, when the eGFR values derived from the other 3 equations were high, the eGFR values obtained from the CKD-EPI equation were systematically lower. The intra-class correlation coefficient (ICCs) and weighted kappa statistics for 5 equations are shown in Supplementary Table S1. The ICCs ranged from 0.68 (MDRD II vs. Mayo) to 0.98 (MDRD II vs. re-expressed MDRD II). When the patients were classified into five categories or two categories, the best agreement was shown between the MDRD II and CKD-EPI equation (weighted kappa = 0.92 and 0.93, respectively) and the worst between the CG and Mayo equation (weighted kappa = 0.75 and 0.51, respectively).

**Figure 2:**
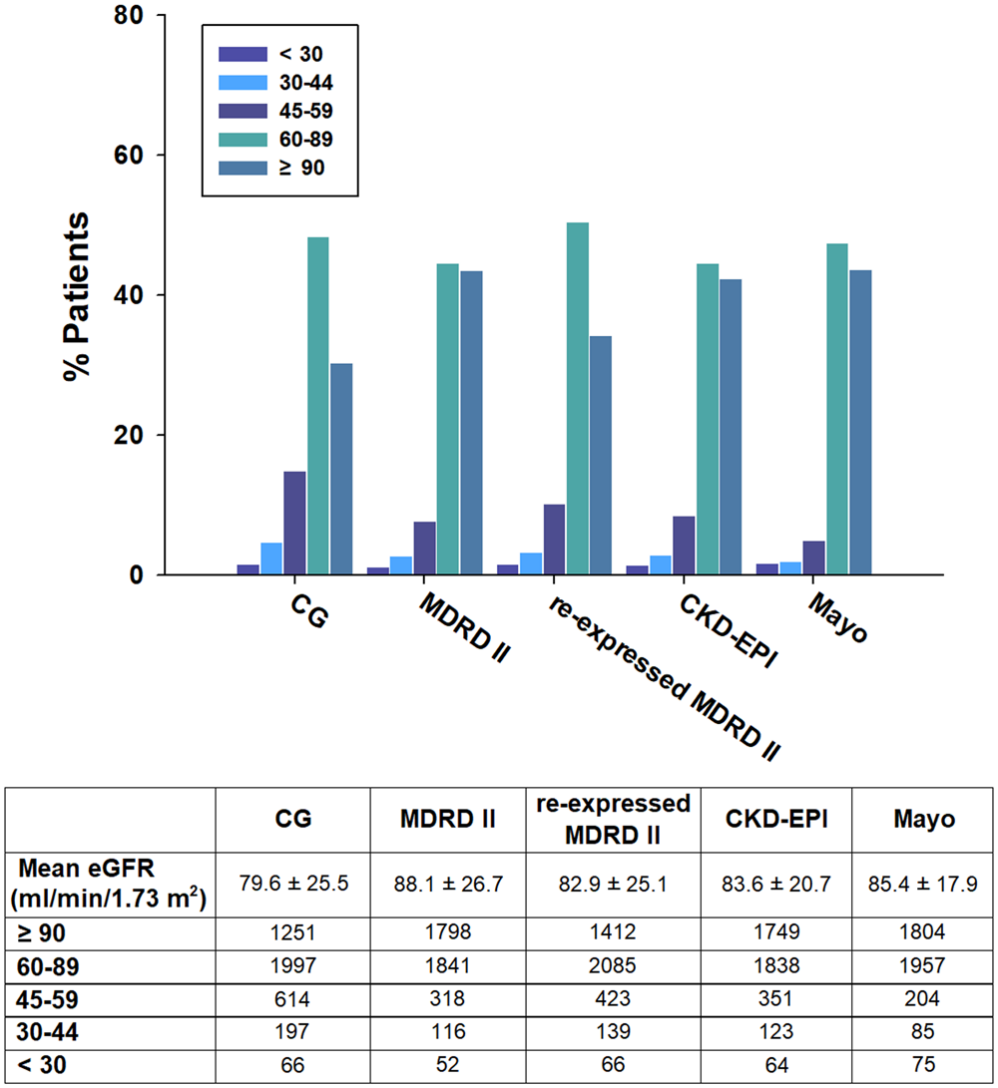
Distribution of patients by preoperative eGFR according to the five equations. eGFR, estimated glomerular filtration rate; CG, Cockroft-Gault; MDRD, Modification of Diet in Renal Disease; CKD-EPI, Chronic Kidney Disease Epidemiology Collaboration; Mayo, Mayo Clinic Quadratic.

The distribution of patients into categories based on eGFRs calculated using the five equations is shown in Fig. 2. The CG equation yielded the lowest mean value (79.6 ± 25.5 ml/min/1.73 m^2^) and MDRD II yielded the highest value (88.1 ± 26.7 ml/min/1.73 m^2^).

The percentage of patients with preoperative eGFR <60 ml/min/1.73 m^2^ differed significantly when eGFR was calculated using the CG (21.3%), MDRD II (11.8%), re-expressed MDRD II (15.2%), CKD-EPI (13.0%), and Mayo (8.8%) equations (*P* < 0.001). Compared with the CKD-EPI equation, the CG equation reclassified a higher proportion of patients as having worse renal function and the Mayo equation reclassified a higher proportion of patients as having better renal function (Supplementary Fig. S2).

The incidence of AKI and KDIGO stage ≥2 decreased from the lower to higher eGFR categories calculated by all five equations (Supplementary Fig. S3). Multivariable analyses showed that lower eGFR, calculated according to each equation, was significantly associated with an increased risk of AKI after cardiovascular surgery. As shown by the adjusted area under the curve (AUC) with 95% confidence interval (CI), eGFR calculated according to all five equations was an effective predictor of postoperative AKI, albeit modest predictive ability of the equations (Table 2). When the AUCs of the equations were compared, the Mayo equation was the most accurate. Net reclassification improvement (NRI) and integrated discrimination improvement (IDI) also showed that the Mayo equation was the most accurate in predicting postoperative AKI (Table 3). In KDIGO stage ≥2 and additional adjustment, similar results were obtained with all equations (Supplementary Tables S2 and S3). However, in KDIGO stage ≥2, the AUC differences between each equation, except for the MDRD II and re-expressed MDRD II equations, were not statistically significant.

**Table 2:** Odds ratio and AUCs for acute kidney injury in preoperative eGFR according to the different equations.

Equation	Acute kidney injury^a^						
	Odds ratio (95% CI)^b^	*P* value	AUC (95% CI)	*P* value 1^c^	*P* value 2^d^	*P* value 3^e^	*P* value 4^f^
CG	0.93 (0.90, 0.96)	<0.001	0.707 (0.689, 0.725)				
MDRD II	0.96 (0.93, 0.99)	0.003	0.704 (0.686, 0.722)	0.011			
re-expressed MDRD II	0.96 (0.93, 0.99)	0.003	0.704 (0.686, 0.722)	0.011	1.000		
CKD-EPI	0.91 (0.87, 0.94)	<0.001	0.708 (0.690, 0.726)	0.192	0.002	0.002	
Mayo	0.87 (0.83, 0.90)	<0.001	0.713 (0.696, 0.731)	<0.001	<0.001	<0.001	<0.001

^a^Adjusted for type of surgery, body mass index, diabetes mellitus, hypertension, dyslipidemia, current smoker, previous myocardial infraction, cerebrovascular disease, peripheral vascular disease, preoperative hematocrit, total bilirubin and albumin levels, left ventricle ejection fraction, preoperative use of β-blocker, calcium channel blocker, insulin, statin, aspirin and clopidogrel.

^b^For each 10 U increase in the scale.

^c^vs. CG; ^d^vs. MDRD II; ^e^vs. re-expressed MDRD II; ^f^vs. CKD-EPI.

AUC, area under the receiver-operating characteristic curve; eGFR, estimated glomerular filtration rate; CI, confidence interval; CG, Cockroft-Gault; MDRD, Modification of Diet in Renal Disease; CKD-EPI, Chronic Kidney Disease Epidemiology Collaboration; Mayo, Mayo Clinic Quadratic.

**Table 3:** Accuracy of each equation in predicting acute kidney injury after cardiovascular surgery.

Equation comparison	Acute kidney injury			
	NRI% (95% CI)	*P* value	IDI% (95% CI)	*P* value
CG vs MDRD II	−20.7 (−27.5, −14.0)	<0.001	−0.3 (−0.4, −0.2)	<0.001
CG vs re-expressed MDRD II	−20.7 (−27.5, −14.0)	<0.001	−0.3 (−0.4, −0.2)	<0.001
CG vs CKD-EPI	10.1 (3.3, 16.9)	0.004	0.2 (0.1, 0.3)	0.002
CG vs Mayo	15.1 (8.3, 21.9)	<0.001	0.6 (0.3, 0.8)	<0.001
MDRD II vs re-expressed MDRD II	—	—	—	—
MDRD II vs CKD-EPI	19.1 (12.3, 26.0)	<0.001	0.4 (0.3, 0.6)	<0.001
MDRD II vs Mayo	16.4 (9.6, 23.2)	<0.001	0.8 (0.6, 1.1)	<0.001
re-expressed MDRD II vs CKD-EPI	19.1 (12.3, 26.0)	<0.001	0.4 (0.3, 0.6)	<0.001
re-expressed MDRD II vs Mayo	16.4 (9.6, 23.2)	<0.001	0.8 (0.6, 1.1)	<0.001
CKD-EPI vs Mayo	13.6 (6.7, 20.4)	<0.001	0.4 (0.2, 0.6)	<0.001

NRI, net reclassification improvement; CI, confidence interval; IDI, integrated discrimination improvement; CG, Cockroft-Gault; MDRD, Modification of Diet in Renal Disease; CKD-EPI, Chronic Kidney Disease Epidemiology Collaboration; Mayo, Mayo Clinic Quadratic.

Comparisons of patients with eGFR ≥60 ml/min/1.73 m^2^ and eGFR <60 ml/min/1.73 m^2^, calculated according to all five equations, showed that the latter group had longer stays in the intensive care unit and in the hospital; and higher rates of postoperative AKI, severe AKI, renal replacement therapy, and in-hospital mortality (Table 4). Moreover, patients with discordant eGFR, as calculated by each of these equations, had poorer outcomes than those with eGFR ≥60 ml/min/1.73 m^2^ in all 5 equations.

**Table 4:** Postoperative outcomes.

Outcomes	Total	Group 1	Group 2	Group 3	*P* value
N	4125	2918	891	316	
Intensive care unit stay (h)	47.0 [39.9–71.0]	46.0 [32.0–65.0]	48.5 [42.5–91.5]	67.3 [43.5–139.3]	<0.001
Hospital stay (d)	9.0 [7.0–14.0]	8.0 [7.0–12.0]	10.0 [7.0–16.0]	11.0 [8.0–21.0]	<0.001
Acute kidney injury	1256 (30.4)	737 (25.3)	346 (38.8)	173 (54.7)	<0.001
KDIGO stage ≥2	417 (10.1)	220 (7.5)	118 (13.2)	79 (25.0)	<0.001
Renal replacement therapy	219 (5.3)	102 (3.5)	65 (7.3)	52 (16.5)	<0.001
In-hospital death	111 (2.7)	45 (1.5)	39 (4.4)	27 (8.5)	<0.001

Data are expressed as number of patients (%) or median [first-third quartiles].

Group 1 = patients with eGFR ≥60 ml/min/1.73 m^2^ according to all five equations, Group 2 = patients with eGFR ≥60 ml/min/1.73 m^2^ with one or more equations and/or eGFR <60 ml/min/1.73 m^2^ depending on the equations used, Group 3 = patients with eGFR <60 ml/min/1.73 m^2^ according to all five equations. KDIGO, Kidney Disease Improving Global Outcomes classification; eGFR, estimated glomerular filtration rate.

## Discussion

This retrospective observational study yielded three main findings. First, despite the relatively good correlation and agreement between eGFRs calculated using all five equations, the proportion of patients classified into different renal function groups varied considerably. Second, although preoperative eGFR calculated using all five equations was a fairly good predictor of AKI after cardiovascular surgery, eGFR calculated using the Mayo equation had the highest accuracy in predicting postoperative AKI. Third, patients who showed alterations from eGFR <60 ml/min/1.73 m^2^ to eGFR ≥60 ml/min/1.73 m^2^, or vice versa, depending on the equations used to calculate eGFR, showed better outcomes than patients with eGFR <60 ml/min/1.73 m^2^, but poorer outcomes than patients with eGFR ≥60 ml/min/1.73 m^2^, according to all five equations.

In contrast to previous studies assessing the effect of preoperative eGFR on postoperative outcomes using just one or two equations^1,2,17^, this study evaluated and compared five equations for calculating eGFR. Although all five equations showed that poorer preoperative renal function tended to increase the risk of postoperative AKI, these equations yielded significant variability in eGFR values. Moreover, the choice of equation affected the stratification of patients by renal function categories, including the dichotomous distinction of eGFR <60 ml/min/1.73 m^2^ versus ≥60 ml/min/1.73 m^2^. For example, the prevalence of eGFR < 60 ml/min/1.73 m^2^ differed nearly 3-fold when calculated by the CG and Mayo equations. This finding is in line with those of previous studies showing that the prevalence of eGFR <60 ml/min/1.73 m^2^ differed significantly, depending on the equation used to calculate GFR^12,13,15,18–20^. One study of 3,270 patients with acute coronary syndrome reported that the prevalence of eGFR <60 ml/min/1.73 m^2^ was highest when calculated by the CG equation (36.1%), intermediate when calculated by the re-expressed MDRD II equation (31.4%), and lowest when calculated by the CKD-EPI equation (30.4%)^13^. Another study of 4,039 patients with ST-elevation myocardial infarction found that the prevalence of eGFR <60 ml/min/1.73 m^2^ was highest when calculated by the CKD-EPI equation (26.5%), intermediate when calculated by the CG (25.7%) and MDRD II (24.3%) equations, and lowest when calculated by the Mayo equation (14.8%)^15^. These equation-dependent differences in the prevalence of eGFR <60 ml/min/1.73 m^2^ may be due to differences in the coefficients and variables included in these equations or differences in study populations. Indeed, the CG equation was developed in a population of male Caucasians with slightly impaired renal function for prediction of creatinine clearance^5^. Because this equation incorporated glomerular and tubular creatinine clearance and its numerator includes weight and 140-age, it may overestimate GFR in younger healthy patients and underestimate GFR in older patients and those with lower body weight^21,22^. Thus, our finding that mean eGFR was lowest and the prevalence of eGFR <60 ml/min/1.73 m^2^ was highest when calculated using the CG equation may be attributed to the relatively older age and lower body mass index of this patient population. However, because underlying GFR was not directly measured by any reference method in this study, the best equation for estimating actual GFR cannot be determined from our results.

It is well established that preoperative renal dysfunction defined as eGFR < 60 ml/min/1.73 m^2^ is linked to both short- and long-term prognosis after surgery^2,3^. However, as shown in our study, the eGFR of the same patient differed significantly depending on the equation employed, indicating a potential for incorrect diagnoses and therapy. For example, it is impossible to diagnose a patient with pre-operative renal dysfunction if eGFR calculated by the re-expressed MDRD II equation is <60 ml/min/1.73 m^2^ and eGFR calculated by the CKD-EPI equation is ≥60 ml/min/1.73 m^2^. Thus, if two different equations with the same sCr concentration yield different eGFR values (i.e., ≥60 ml/min/1.73 m^2^ from one equation and <60 ml/min/1.73 m^2^ from the other equation), it may be necessary to check GFR directly using any reference method. On the other hand, if this criterion is incorporated into preoperative assessments, this group of patients would be identified as being at increased risk of poor postoperative outcomes. Indeed, our results indicated that patients with discordant eGFR, as calculated by these equations, showed poorer outcomes than patients with eGFR ≥60 ml/min/1.73 m^2^ calculated by all five equations. Thus, our finding suggests that estimating GFR using two or more equations may help in the risk stratification of surgical patients, with patients having equation-dependent preoperative eGFR ≥60 ml/min/1.73 m^2^ or <60 ml/min/1.73 m^2^ requiring closer monitoring and more intensive care during the perioperative period to improve outcomes. Although this will requires validation in a larger study population, it may be clinically meaningful, as this finding is in line with those of previous studies showing that patient whose eGFR estimated as ≥60 ml/min/1.73 m^2^ using one equation shifted to <60 ml/min/1.73 m^2^ using another equation had mortality rates that were intermediate between those with eGFR <60 ml/min/1.73 m^2^ and those with eGFR ≥60 ml/min/1.73 m^2^, according to all five equations in a broad spectrum of acute coronary syndrome patients^15^.

The KDIGO clinical practice guidelines recommend using the CKD-EPI equation, based on sCr concentration, for the detection, determination of severity, and treatment of kidney disease^10^. However, it is still unclear whether it is the best equation for predicting prognosis. Indeed, although several studies compared the ability of eGFR equations to predict prognosis in patients with various medical conditions, including heart failure and acute coronary syndrome, the results varied^11–16,18^. For example, a meta-analysis comparing the risk implications of the CKD-EPI and MDRD equations in 1,130,472 adults from 25 general populations found that the CKD-EPI equation more accurately classified risks of mortality and end-stage renal disease^18^. However, studies comparing eGFR equations in patients with heart failure and acute coronary syndrome found that the CG equation was better able to predict the risks of mortality and bleeding complications^12,13,16^. Additionally, a recent study comparing the CG, MDRD, CKD-EPI, Mayo, and inulin clearance-based eGFR equations in 8,726 patients with acute coronary syndrome found that the Mayo equation was better at predicting mortality than the other four equations^15^. In our study, the Mayo equation was more accurate in predicting postoperative AKI than other four equations. It is not clear why Mayo equation, the rarely used equation nowadays, was slightly more accurate in predicting postoperative AKI than other equations. A possible explanation for the better predictive value of Mayo equation over other equations is that it tends to show relatively better eGFR values during reclassification. Methodologically, both NRI and IDI showed better predictability when calculating higher eGFR value in patients without AKI and when calculating lower eGFR values in patients with AKI. In this study cohort, the overall incidence of AKI was approximately 30%, and the values calculated were higher for patients without than with AKI, suggesting that the Mayo equation showed good predictive power. Another potential explanation is that differences in the population that is evaluated may contribute to which equation is better as a predictor. The MDRD equation was developed in patients with chronic kidney disease which may systematically underestimate GFR at higher values^7,9^. By contrast, the CKD-EPI and Mayo equations were developed in patients with normal renal function and chronic kidney disease resulting in better estimations of GFR in populations with preserved renal function or nonsignificant renal dysfunction^8,9,23^. Given that the majority of our cohorts had a high pre-operative eGFR in all five equations, the poorer predictive ability of the MDRD equation and the better predictive ability of the CKD-EPI and Mayo equations could be partly explained.

eGFR is a cheap, practical, and fairly reliable method to evaluate renal function in clinical practice^4^. Our results suggest that preoperative eGFR may serve not only to assess renal function, but also to estimate the risk of postoperative AKI. Indeed, regardless of how it is calculated, preoperative eGFR <60 ml/min/1.73 m^2^ is an important predictor of postoperative AKI. Thus, our results suggest that preoperative assessment of renal function using eGFR in patients undergoing cardiovascular surgery may be needed to identify those at high risk of AKI. Additionally, in order to improve short- and long-term outcomes, renal protective strategies such as discontinuation of nephrotoxic agents or prevention of major hemodynamic events should be implemented early in patients with preoperative eGFR <60 ml/min/1.73 m^2^.

This study has several limitations. First, because of the retrospective and observational nature of this study, despite including many variables in our analyses, other hidden or unknown factors including fluid therapy or hemodynamics during perioperative period may have influenced our results^24,25^. Second, we did not assess outcomes other than AKI after cardiovascular surgery. Thus, we cannot exclude the possibility that differences in outcomes may have contributed to the predictive ability of the eGFR equations. In fact, the Mayo equation was not superior to the CG and CKD-EPI equations for severe form of AKI (KDIGO stage ≥2) prediction in this cohort. Third, this study did not include data on directly measured GFR values using gold standard methods or on markers of kidney damage, including albuminuria, thus precluding a definitive conclusion about the association between actual preoperative renal function and postoperative AKI. In addition, this study assessed the ability of calculated eGFRs to predict postoperative AKI, not their accuracy in determining GFR. Therefore, although the Mayo equation was most accurate in predicting postoperative AKI, this does not mean that it should be used to replace other equations to estimate renal function. Indeed, the Mayo equation has several drawbacks, such as difficulty in establishing its extensive generalization and use, as it was derived from relatively few patients, and has shown conflicting results in patients with diabetes^8,23,26–28^. Moreover, this equation has not been evaluated in Asian populations. Indeed, in daily clinical practice, the CKD-EPI and CG equations, rather than the Mayo equation, are most commonly used to calculate eGFR or creatinine clearance. Thus, despite the fact that the Mayo equation was slightly more accurate in terms of predicting the risk of postoperative AKI in patients undergoing cardiovascular surgery, it should be emphasized that our results do not imply that the Mayo equation should be used for GFR estimation and that this equation is not recommended by updated guidelines. Finally, this was a single centre study conducted almost exclusively in East Asian patients. Because all five equations tested in this study can be influenced by race and ethnicity, care should be taken when generalizing these results to centres with different patient profiles.

In conclusion, preoperatively decreased eGFR, as determined using all five equations, was an independent predictor of postoperative AKI in patients undergoing cardiovascular surgery. Although the differences in predictive ability between equations were very small, the Mayo equation was slightly more accurate than the other equations in predicting AKI after cardiovascular surgery.

## Methods

### Study design and subjects

This retrospective cohort included all patients aged ≥20 years who underwent elective cardiovascular surgery at Asan Medical Centre, a tertiary hospital in Seoul, South Korea, between January 2010 and July 2015. Clinical information, including demographic data, comorbidities, laboratory data, medication use, perioperative management, and morbidity and mortality, was acquired from the Cardiovascular Surgery and Anaesthesia Database of Asan Medical Centre and from a retrospective review of its computerized patient record system (Asan Medical Centre Information System Electronic Medical Record)^29^. Patients lacking information on preoperative sCr concentration; those who underwent preoperative dialysis, endovascular aortic repair surgery, or preoperative implantation of an intra-aortic balloon pump or ventricular assist device support; and patients with a history of organ transplantation or nephrectomy were excluded. This study was performed in agreement with the Strengthening the Reporting of Observational Studies in Epidemiology guidelines^30^, and was approved by the Institutional Review Board of Asan Medical Centre (AMC IRB 2017-0336), which waived the requirement for informed consent.

### Calculation of eGFR to assess renal function

eGFR was calculated using five different equations, based on a single preoperative determination of sCr concentration, measured closest to the time of surgery (but within 30 days of surgery), using the kinetic Jaffe method (Cobas^®^ 8000 modular analyser series; Roche Diagnostics GmbH, Vienna, Austria). The five equations used to calculate eGFR were as follows:

1. CG equation^5^: eGFR = [(140 − Age) × Weight/(72 × sCr)] × (0.85 if female)
2. MDRD II equation^6^: eGFR = 186 × sCr^−1.154^ × Age^−0.203^ × (0.742 if female) × (1.210 if African-American)
3. re-expressed MDRD II equation^7^: eGFR = 175 × sCr^−1.154^ × Age^−0.203^ × (0.742 if female) × (1.210 if African-American)
4. CKD-EPI equation^9^: eGFR = 141 × min(sCr/κ, 1)^α^ × max(sCr/κ, 1)^−1.209^ × 0.993^age^ × 1.018 (if female) × 1.159 (if Black), where κ is 0.7 for females and 0.9 for males, α is −0.329 for females and −0.411 for males, min indicates the minimum of sCr/κ or 1, and max indicates the maximum of sCr/κ or 1.
5. Mayo equation^8^: eGFR = exp [1.911 + 5.249/sCr − 2.114/sCr^2^ − 0.00686 × Age − (0.205 if female)], if sCr < 0.8 mg/dl then sCr = 0.8.

The CG equation was adjusted for body surface area. Body surface area is calculated by the following DuBois equation: body surface area = 0.20247 × Weight^0.425^ × Height^0.725^ ^31^. Units are weight (kg), height (m), age (year), and sCr (mg/dl).

Preoperative eGFRs calculated by each equation were classified into the five categories of the KDIGO classification: ≥90, 89–60, 59–45, 44–30, and <30 ml/min/1.73 m^2^ ^10^.

### End points

The primary study outcome was the incidence of postoperative AKI, defined by KDIGO criteria as a ≥0.3 mg/dl increase in sCr concentration within 48 h of surgery or a ≥1.5-fold increase in sCr from baseline within 7 days of surgery^32^. Patients who met the KDIGO criteria were classified as “AKI”, whereas those who did not were classified as “no AKI”. Patients with AKI were staged according to the maximum KDIGO criteria, with stage 1 defined as sCr increases of ≥0.3 mg/dl or ≥1.5 times baseline; stage 2 as sCr increases 2.0–2.9 times baseline; and stage 3 as sCr increases ≥3.0 times baseline or ≥4.0 mg/dl, or the initiation of renal replacement therapy. AKI was evaluated based on the highest sCr concentration measured within 7 days after surgery. AKI was not diagnosed based on urine output due to incomplete recording and the effects of administered diuretics. Secondary outcomes included the incidence of KDIGO stage ≥2 after surgery.

### Statistical analysis

All data manipulations and statistical analyses were performed using R version 3.1.2 (R Foundation for Statistical Computing, Vienna, Austria) and IBM SPSS Statistics 21.0 (IBM Corp., Armonk, NY, USA). The sample size corresponded to all patients included in the study, with no *a priori* power analysis conducted. Categorical variables were expressed as numbers and percentages, and continuous variables were expressed as mean ± standard deviation or median and interquartile range.

Correlations between eGFR calculated using the CKD-EPI equation and the four other equations were assessed by Pearson correlation analyses. To assess the agreement between the values of each equation, Bland-Altman analyses and ICCs was used for continuous variables and weighted kappa statistic with squared weights was used for categorical variables.

Multivariable logistic regression analyses were performed to evaluate the independent associations between the preoperative eGFR and postoperative renal outcomes. All predictive preoperative clinical and surgical variables in Table 1 were assessed independently, and variables with a *P* value <0.05 in univariate analyses were included in the multivariable analyses. Adjusted odds ratio and 95% CI for the logistic regression were calculated. The Hosmer and Lemeshow goodness-of-fit test was used to measure calibration, or the ability of the model to make unbiased estimates of outcome. For sensitivity analysis, the effects of additional adjustment for potential confounding variables of known risk factors for AKI were also explored.

Receiver operating characteristic analyses were performed, and the results were presented as adjusted AUCs with 95% CIs, to evaluate the discriminatory ability and the ability of preoperative eGFR, calculated using the five equations, to predict postoperative renal outcomes. The AUCs of the models were compared as described^33^. The continuous NRI was determined to assess the ability of the different equations to improve risk prediction of postoperative renal outcomes. The IDI for each eGFR was also calculated. The IDI evaluates the difference between the integrated sensitivity gain and the integrated specificity loss due to the addition of eGFR to the prognostic model.

All reported *P* values were two-sided, and *P* values < 0.05 were considered statistically significant.

## Supplementary information

Supplementary material

## Data Availability

All data are available from the corresponding author upon reasonable request.
