# Supplementary material for: Comparison of five glomerular filtration rate estimating equations as predictors of acute kidney injury after cardiovascular surgery
Source: Sci Rep. 2019 Jul 30;9:11072. doi: 10.1038/s41598-019-47559-w (PMC6667489; doi:10.1038/s41598-019-47559-w)
Supplement: Supplementary file 1 — Supplementary material [file 41598_2019_47559_MOESM1_ESM.pdf]

-Supplementary Materials-

Comparison of five glomerular filtration rate estimating equations as predictors of acute kidney injury after cardiovascular surgery

Jun-Young Jo, M.D., Seung Ah Ryu, M.D., Jong-Il Kim, M.D., Eun-Ho Lee, M.D, Ph.D., In-Cheol Choi, M.D.,Ph.D.

Table of Contents

Table S1. Agreements between estimated glomerular filtration rates calculated by each pair of equations

Table S2. Odds ratios and AUCs for KDIGO stage ≥ 2 based on preoperative eGFR calculated by the five different equations

Table S3. Odds ratio and AUCs for acute kidney injury in additional adjustment model

Figure S1. Correlation and Bland-Altman analysis of eGFRs calculated by each of the four equations when compared with the CKD-EPI equation. CG, Cockroft-Gault; MDRD, Modification of Diet in Renal Disease; CKD-EPI, Chronic Kidney Disease Epidemiology Collaboration.

Figure S2. Reclassification of CKD-EPI-based eGFR categories based on other equations in the total cohort. CKD-EPI, Chronic Kidney Disease Epidemiology Collaboration; eGFR, estimated glomerular filtration rate; CG, Cockcroft-Gault; MDRD, Modification of Diet in Renal Disease.

Figure S3. AKI (A) and KDIGO stage ≥ 2 (B) after cardiovascular surgery based on preoperative eGFR calculated by the five different equations. AKI, acute kidney injury; CG, Cockcroft-Gault; MDRD, Modification of Diet in Renal Disease; CKD-EPI, Chronic Kidney Disease Epidemiology Collaboration; eGFR, estimated glomerular filtration rate.

Table S1. Agreements between estimated glomerular filtration rates calculated by each pair of equations

Equation	ICC (95% CI)	Weighted kappa (95% CI)[a]	Weighted kappa (95% CI)[b]
CG vs MDRD II	0.84 (0.62, 0.92)	0.78 (0.65, 0.85)	0.61 (0.55, 0.67)
CG vs re-expressed MDRD II	0.88 (0.86, 0.90)	0.81 (0.79, 0.83)	0.69 (0.66, 0.72)
CG vs CKD-EPI	0.88 (0.83, 0.91)	0.83 (0.73, 0.88)	0.67 (0.62, 0.72)
CG vs Mayo	0.73 (0.65, 0.79)	0.75 (0.60, 0.83)	0.51 (0.41, 0.58)
MDRD II vs re-expressed MDRD II	0.98 (0.08, 1.00)	0.90 (0.85, 0.93)	0.85 (0.84, 0.87)
MDRD II vs CKD-EPI	0.87 (0.82, 0.91)	0.92 (0.92, 0.93)	0.93 (0.92, 0.93)
MDRD II vs Mayo	0.68 (0.66, 0.70)	0.79 (0.77, 0.80)	0.77 (0.76, 0.79)
re-expressed MDRD II vs CKD-EPI	0.90 (0.89, 0.91)	0.89 (0.86, 0.91)	0.91 (0.90, 0.91)
re-expressed MDRD II vs Mayo	0.70 (0.68, 0.72)	0.74 (0.69, 0.78)	0.70 (0.66, 0.74)
CKD-EPI vs Mayo	0.90 (0.88, 0.91)	0.85 (0.84, 0.86)	0.78 (0.76, 0.80)

[a] Five categories: ≥ 90, 89–60, 59–45, 44–30, and < 30 ml min^{-1} 1.73 m^{-2}.

[b] Two categories: ≥ 60 and < 60 ml min^{-1} 1.73 m^{-2}.

ICC, intra-class correlation coefficient; CI, confidence interval; CG, Cockroft-Gault; MDRD, Modification of Diet in Renal Disease; CKD-EPI, Chronic Kidney Disease Epidemiology Collaboration; Mayo, Mayo Clinic Quadratic.

Table S2. Odds ratios and AUCs for KDIGO stage ≥ 2 based on preoperative eGFR calculated by the five different equations

	KDIGO stage ≥ 2[a]						
Equation	Odds ratio (95% CI)[b]	*P* value	AUC (95% CI)	*P* value 1[c]	*P* value 2[d]	*P* value 3[e]	*P* value 4[f]
CG	0.94 (0.90, 0.98)	0.002	0.756 (0.731, 0.781)				
MDRD II	0.96 (0.92, 0.99)	0.025	0.755 (0.730, 0.779)	0.278			
re-expressed MDRD II	0.96 (0.92, 0.99)	0.025	0.755 (0.730, 0.779)	0.278	1.000		
CKD-EPI	0.91 (0.87, 0.96)	< 0.001	0.757 (0.733, 0.782)	0.271	0.085	0.085	
Mayo	0.89 (0.85, 0.94)	< 0.001	0.760 (0.735, 0.784)	0.087	0.024	0.024	0.129

[a] adjusted for type of surgery, body mass index, diabetes mellitus, hypertension, dyslipidaemia, current smoker, previous myocardial infarction, cerebrovascular disease, peripheral vascular disease, preoperative haematocrit, total bilirubin, and albumin levels, left ventricle ejection fraction, and preoperative use of β-blockers, calcium channel blockers, insulin, statins, aspirin, and clopidogrel.

[b]: for each 10 U increase in the scale.

[c] vs. CG; [d] vs. MDRD II; [e] vs. re-expressed MDRD II; [f] vs. CKD-EPI.

AUC, area under the receiver operating characteristic curve; eGFR, estimated glomerular filtration rate; CI, confidence interval; CG, Cockroft-Gault; MDRD, Modification of Diet in Renal Disease; CKD-EPI, Chronic Kidney Disease Epidemiology Collaboration; Mayo, Mayo Clinic Quadratic.

Table S3. Odds ratio and AUCs for acute kidney injury in additional adjustment model

	Acute kidney injury[a]						
Equation	Odds ratio (95% CI)[b]	*P* value	AUC (95% CI)	*P* value 1[c]	*P* value 2[d]	*P* value 3[e]	*P* value 4[f]
CG	0.96 (0.93, 0.99)	0.037	0.734 (0.718, 0.751)				
MDRD II	0.99 (0.96, 1.01)	0.307	0.733 (0.717, 0.750)	0.080			
re-expressed MDRD II	0.98 (0.96, 1.02)	0.307	0.733 (0.717, 0.750)	0.080	1.000		
CKD-EPI	0.95 (0.91, 0.99)	0.008	0.735 (0.718, 0.751)	0.477	0.055	0.055	
Mayo	0.91 (0.87, 0.96)	< 0.001	0.737 (0.720, 0.753)	0.029	0.005	0.005	0.008

[a] adjusted for type of surgery, age, body mass index, diabetes mellitus, hypertension, dyslipidemia, current smoker, previous myocardial infraction, cerebrovascular disease, peripheral vascular disease, preoperative anemia, total bilirubin and albumin levels, left ventricle ejection fraction, preoperative use of β-blocker, calcium channel blocker, insulin, statin, aspirin, clopidogrel, cardiopulmonary bypass time, and intraoperative use of packed red blood cell.

[b]: for each 10 U increase in the scale

[c] vs. CG; [d] vs. MDRD II; [e] vs. re-expressed MDRD II; [f] vs. CKD-EPI.

AUC, area under the receiver-operating characteristic curve; eGFR, estimated glomerular filtration rate; CI, confidence interval; CG, Cockroft-Gault; MDRD, Modification of Diet in Renal Disease; CKD-EPI, Chronic Kidney Disease Epidemiology Collaboration; Mayo, Mayo Clinic Quadratic.

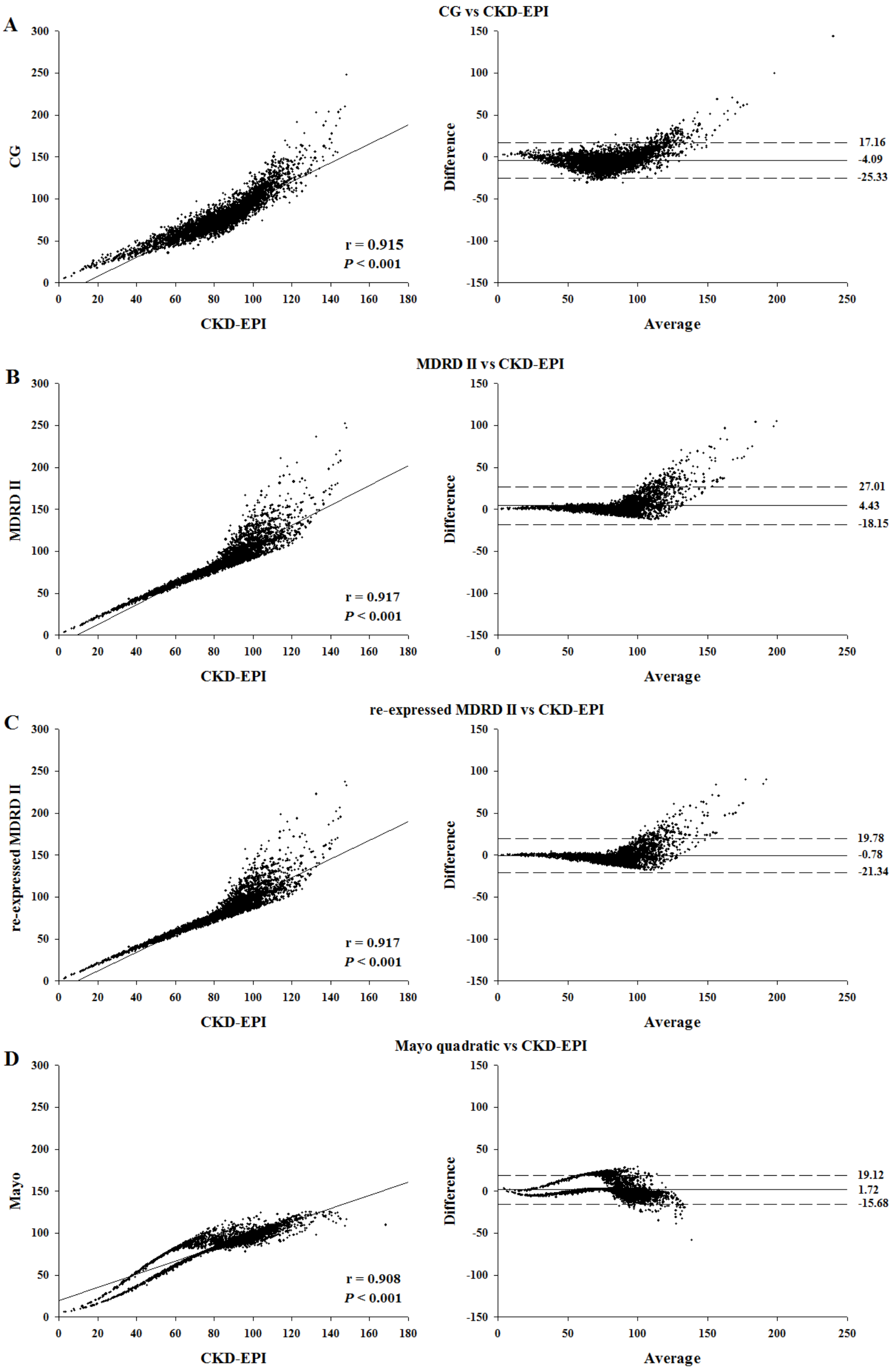

Figure S1. Correlation and Bland-Altman analysis of eGFRs calculated by each of the four equations when compared with the CKD-EPI equation. CG, Cockroft-Gault; MDRD, Modification of Diet in Renal Disease; CKD-EPI, Chronic Kidney Disease Epidemiology Collaboration.

CG eGFR category ml/min/1.73 m²	CKD-EPI eGFR category ml/min/1.73 m²					
	< 30	30-44	45-59	60-89	≥ 90	Total
**< 30**	56	10				66
**30-44**	8	103	79	7		197
**45-59**		10	247	357		614
**60-89**			25	1428	544	1997
**≥ 90**				46	1205	1251
**Total**	64	123	351	1838	1749	4125

MDRD II eGFR category ml/min/1.73 m²	CKD-EPI eGFR category ml/min/1.73 m²					
	< 30	30-44	45-59	60-89	≥ 90	Total
**< 30**	51	1				52
**30-44**	13	101	2			116
**45-59**		21	290	7		318
**60-89**			59	1634	148	1841
**≥ 90**				197	1601	1798
**Total**	64	123	351	1838	1749	4125

re-expressed MDRD II eGFR category ml/min/1.73 m²	CKD-EPI eGFR category ml/min/1.73 m²					
	< 30	30-44	45-59	60-89	≥ 90	Total
**< 30**	63	3				66
**30-44**	1	119	19			139
**45-59**		1	330	92		423
**60-89**			2	1638	445	2085
**≥ 90**				108	1304	1412
**Total**	64	123	351	1838	1749	4125

Mayo eGFR category ml/min/1.73 m²	CKD-EPI eGFR category ml/min/1.73 m²					
	< 30	30-44	45-59	60-89	≥ 90	Total
**< 30**	58	17				75
**30-44**	6	60	19			85
**45-59**		42	160	2		204
**60-89**		4	172	1531	250	1957
**≥ 90**				305	1499	1804
**Total**	64	123	351	1838	1749	4125

Figure S2. Reclassification of CKD-EPI-based eGFR categories based on other equations in the total cohort. CKD-EPI, Chronic Kidney Disease Epidemiology Collaboration; eGFR, estimated glomerular filtration rate; CG, Cockcroft-Gault; MDRD, Modification of Diet in Renal Disease.

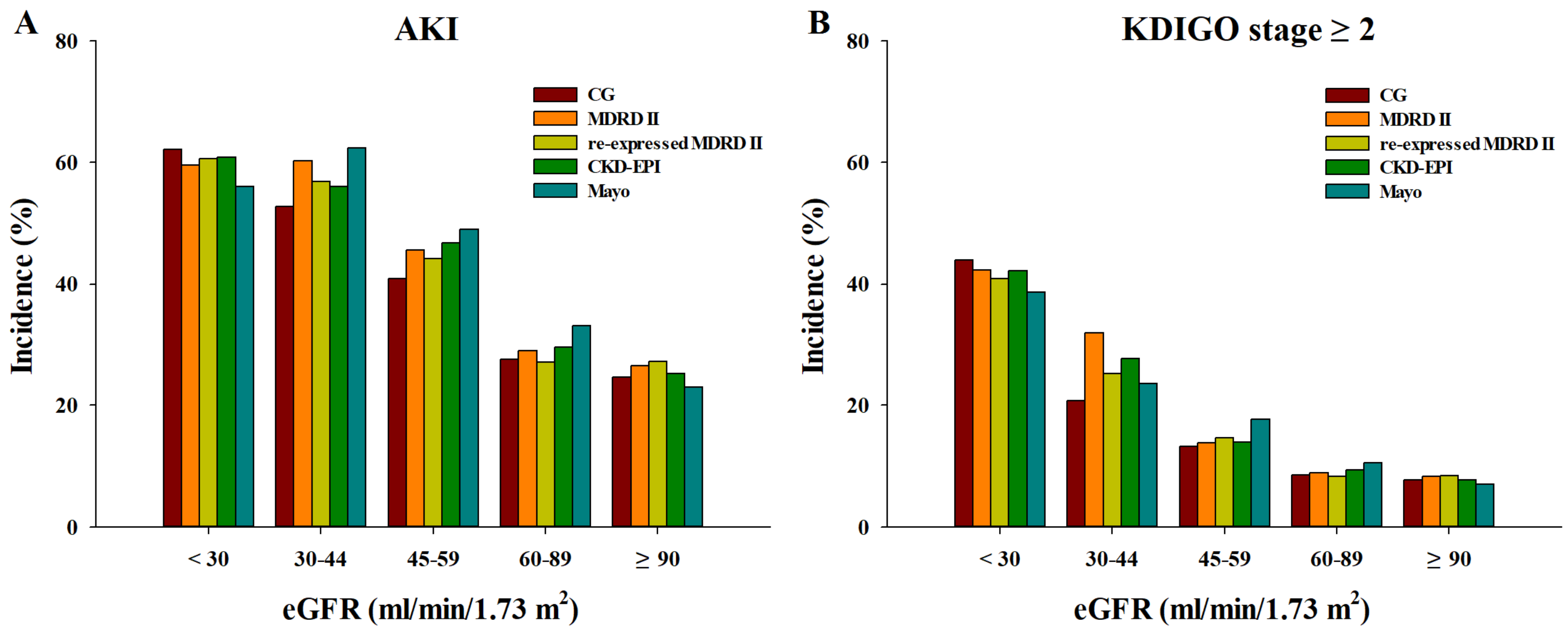

Figure S3. AKI (A) and severe AKI (B) after cardiovascular surgery based on preoperative eGFR calculated by the five different equations. AKI, acute kidney injury; CG, Cockcroft-Gault; MDRD, Modification of Diet in Renal Disease; CKD-EPI, Chronic Kidney Disease Epidemiology Collaboration; eGFR, estimated glomerular filtration rate.
